# Evaluation of Shear Bond Strength of Lithium Disilicate Veneers Using Pre-heated Resin Composite With Two Conventional Resin Cements: An In Vitro Study

**DOI:** 10.7759/cureus.74479

**Published:** 2024-11-26

**Authors:** Ghalia Akyle, Hassan Achour

**Affiliations:** 1 Department of Aesthetic Dentistry, Damascus University, Damascus, SYR; 2 Department of Endodontic and Operative Dentistry, Damascus University, Damascus, SYR

**Keywords:** dual-cured resin cement, lithium disilicate veneers, pre-heated resin composite, resin cements, shear bond strength

## Abstract

Objectives

This study aimed to compare the shear bond strength of three resin cements (light-cured resin cement, pre-heated composite resin, and dual-cured self-adhesive resin cement) when bonding to lithium disilicate discs.

Materials and methods

Thirty-six discs made of lithium disilicate were fabricated and etched with 9.5% (HF), and 36 human premolars were collected and immersed in the acrylic molds, then randomly divided into three equal groups (n = 12): Group 1: light-cured resin cement, Group 2: pre-heated resin composite, and Group 3: dual-cured resin cement. The resin composite was heated between 55°C and 65°C by a heater device. The shear bond strength test was performed using the general mechanical testing device. The data were analyzed using two-way ANOVA and Bonferroni tests (p < 0.05).

Results

The highest shear bond strength was demonstrated by the light-cured resin cement group (26.61 ± 5.16 Mpa), followed by the dual-cured resin cement group (17.76 ± 4.67 Mpa), and the least by the pre-heated composite resin group (15.58 ± 3.36 MPa). The shear bond strength in the light-cured resin cement group was significantly higher than the dual-cured resin cement and pre-heated composite resin groups.

Conclusion

The light-cured resin cement has higher shear bond strength when compared to pre-heated resin composite and dual-cured resin cement with a self-etch system. Although pre-heating composite resins may increase its mechanical proprieties and make it suitable for luting ceramics it may not increase bond strength.

## Introduction

Due to the importance of the smile and the widespread use of porcelain veneers, dental treatments that aim to redesign the smile have become routine treatments performed in most dental clinics [1].

The development of bonding systems and resins used in adhering and the increasing interest of patients in aesthetic aspects has led to a great renaissance in cosmetic dentistry materials [2]. Glass porcelain reinforced with lithium disilicate crystals was used in the manufacture of porcelain veneers for its high aesthetic and mechanical properties, which in turn led to improving the smile and treating some minor dental problems [3].

The clinical success of porcelain veneers made of (IPS e.max) came from their ability to bond to the teeth structures, in addition to the various properties of (IPS e.max): biocompatibility, thermal expansion coefficient similar to that of the teeth structure [4], durability, high resistance to corrosion, excellent compatibility with the optical properties of the teeth structure, color stability, the adhesion between luting agents and the prepared teeth [5].

Porcelain veneers are considered to be very conservative, allowing preparation within the enamel only, which ensures a stronger bond than bonding with dentin [6].

There has been a significant development in the resins materials used in bonding ceramic veneers, whether in terms of their type, mechanical properties, or chemical composition [7], especially since the concept of bonding ceramics using pre-heated composite resins has recently developed due to the change in some of its physical properties [8], including when heated it becomes less viscous than traditional resins [9], pre-heated composite resins is a reliable restorative material due to its good wear resistance, excellent esthetics as well as its large amount of fillers content that decreases its polymerization shrinkage and increases its mechanical properties [10], which makes it suitable for bonding ceramic veneers similar to resin cement [11].

This study aimed to compare the shear bond strength (SBS) of the pre-heated resin composite, light-cured resin cement, and dual-cured self-etch resin cement bonded to lithium‑disilicate ceramic veneers.

## Materials and methods

This is an in vitro study to compare the shear bond strength between three luting resin cements (light-cured resin cement, pre-heated resin composite, and dual-cured self-adhesive resin cement) in extracted premolars for orthodontic reasons. The study protocol was approved by the Scientific Research and Postgraduate Board of Damascus University, Damascus University Ethics Committee, Damascus, Syria (IRB No. UDDS-128-11112023/SRC-1600).

The sample size was determined using a sample size calculation program (PS Power and Sample Size Calculation Program, Version 3.0.43, Vanderbilt University Medical Center (VUMC), Nashville, TN). The sample size required to detect a significant difference was 36 teeth (effect size 0.65, 90% power, and two-sided 5% significance level).

**Figure 1:**
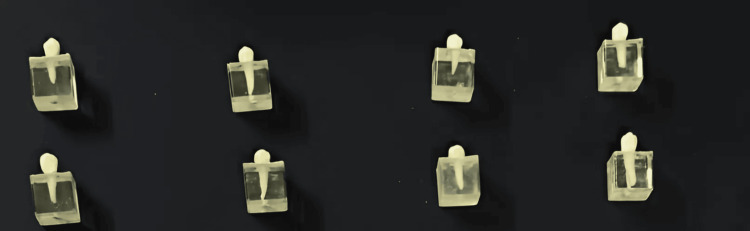
The study sample in acrylic molds.

A total number of 36 human-extracted premolars were selected for this study. The selected teeth were extracted for orthodontic reasons. Teeth were caries-free, did not undergo previous restorative treatment, and had no cracks. The teeth roots were immersed in the acrylic molds, and the teeth crowns were kept elevated above the acrylic molds (Figure 1).

Thirty-six discs from wax with a thickness of 2 mm and a diameter of 3.5 mm were machined, then they were placed in the injection crucible, (from Ivoclar Vivadent) according to the manufacturer's instructions. Then the crucible was placed in the heating oven at a temperature of 850 for 45 minutes to evaporate the resin and after heating to a temperature of 920, the ceramic compressed materials were injected using the aluminum piston according to the injection program followed by the company at a pressure of 5 bars and then left to cool at room temperature. The ceramic discs were sandblasted followed by finishing and polishing then cleaned with an ultrasonic device for five minutes.

Thirty-six ceramic disks made of lithium disilicate ingots (IPS e.max Press, Ivoclar Vivadent, Schaan, Liechtenstein), were prepared in the above ways. The teeth of the study sample were randomly split into three groups (n=12): Group 1, which was cemented by Choice2 Light-Cured Veneer Cement (Choice 2 Veneer Cement, Bisco, Schaumburg, IL); Group 2, which was cemented by Aelite Aesthetic Light-Cured Reinforced Nanofil Composite (Aelite Aesthetic Enamel, Bisco, Schaumburg, IL); and Group 3, which was cemented by TheraCem Dual-Cured, Self-Adhesive Resin Cement in a self-etch system (TheraCem Self-Adhesive Resin Cement, Bisco, Schaumburg, IL).

Sample preparation

**Figure 2:**
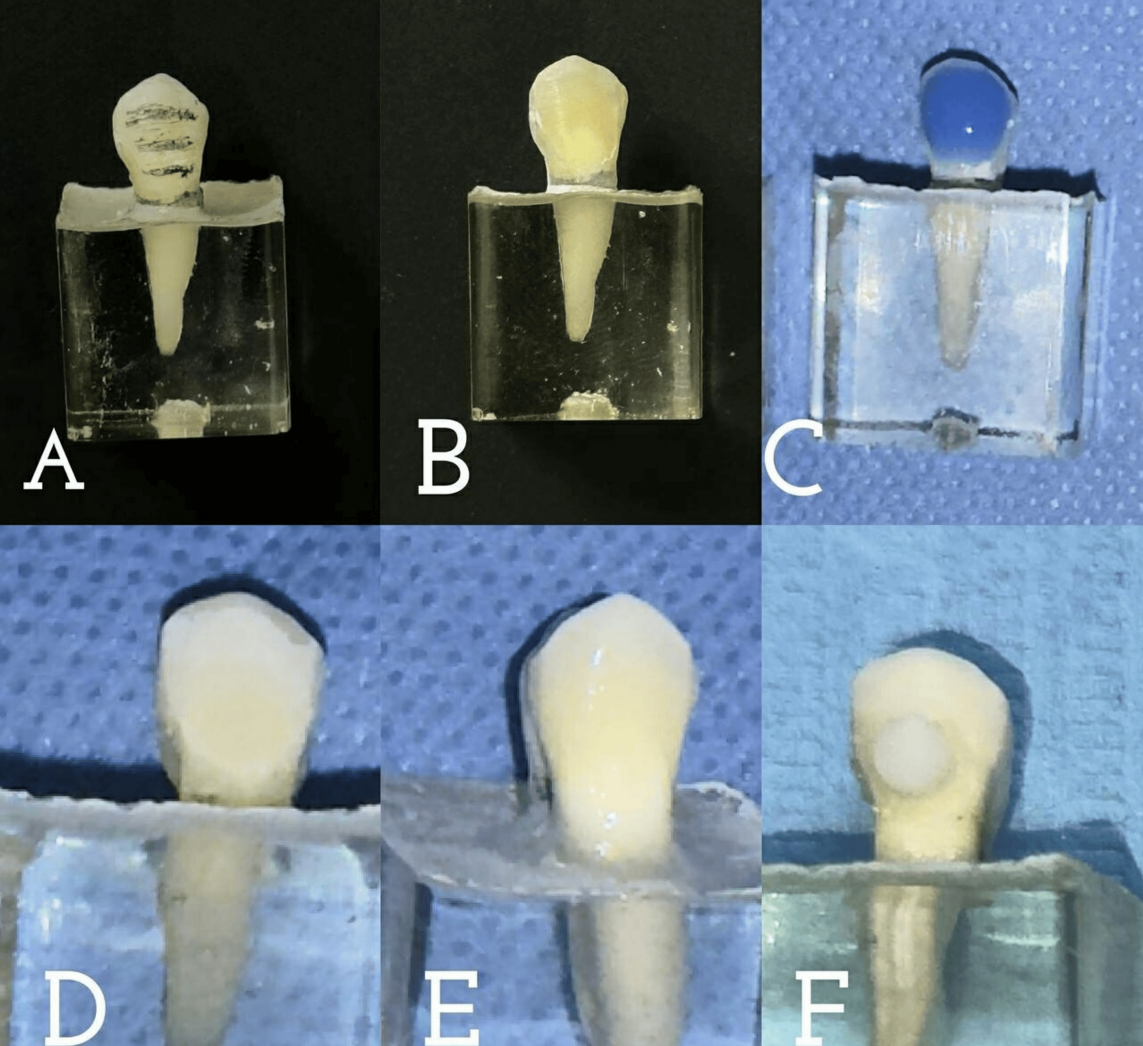
Tooth preparation and veneers application steps. (A, B) Buccal surface preparation, (C) etching the buccal surface with 37% phosphoric acid, (D) buccal surface appearance after etching, (E) buccal surface after bonding, and (F) bonding IPS e.max disc.

Buccal surfaces of the sample teeth were prepared within the enamel only by a single operator, the preparation depth in the buccal surface ranged from 0.4 to 0.7 (Figures 2A, 2B). To control the amount of preparation and determine the preparation depth, the LVS-1 depth cutter burr was used.

In Group 1 and Group 2 teeth surfaces were etched for 30 seconds with 37% phosphoric acid (Meta, South Korea) (Figure 2C), rinsed, gently dried (Figure 2D), and then bond was applied (Figure 2E). In Group 3 teeth surfaces weren’t etched but only a bond was applied.

All the ceramic discs were etched for 20 seconds with 9.5% HF (hydrofluoric acid gel) (Porcelain Etchant™, Bisco, Schaumburg, IL) then were rinsed, dried, and the discs were coated with a single coat of silane (Porcelain Primer™, Bisco, Schaumburg, IL) (Figure 2F). The specimens were stored in distilled water in plastic containers and placed in a thermal incubator, at 37°C for seven days to simulate the oral environment.

Shear bond strength evaluation

**Figure 3:**
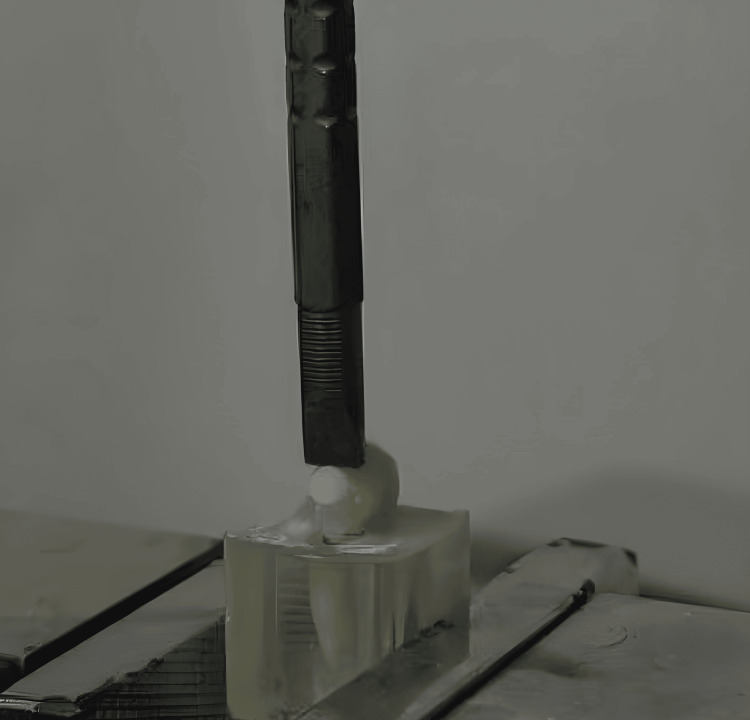
Specimen that has undergone shear bond strength test.

The shear bond strength (SBS) test was performed using the general mechanical testing device (Testometric M350-10KN) located at the Industrial Research Testing Center of the Ministry of Industry in Damascus. The sample was subjected to constant loading forces until the ceramic disc was disengaged from the tooth. The head of the device was parallel to the buccal surface of the tooth and applied so that it was completely between the ceramic disc and the tooth surface at a crosshead speed of 0.5 mm/min (Figure 3). The load required to debonding was recorded in Newton. The load at failure was divided by the bonding area to express the bond strength in MPa: τ = P/ πr2, where τ = microshear bond strength (MPa), P = load at failure (N), π =3.14 and r = radius of microcylinder (mm).

## Results

The study sample consisted of 36 extracted premolars. To compare the shear bond strength values of three resin luting agents used in this study, the Analysis of Variance test (ANOVA) was used. The highest shear bond strength was demonstrated by the light-cured resin cement group (26.61 ± 5.16 Mpa), followed by the dual-cured resin cement group (17.76 ± 4.67 Mpa), and then the pre-heated resin composite group (15.58 ± 3.36 MPa). One-way ANOVA test showed a significant difference between groups (p< 0.05; Table 1).

**Table 1:** One-way ANOVA test. ^* ^Statistically significant at 0.05.

Groups	N	Mean (Mpa)	SD (Mpa)	Min	Max	p-value
Light-cured resin cement	12	26.61	5.16	20.22	37.33	0.000^*^
pre-heated resin composite	12	15.58	3.36	11.70	21.38	
Dual-cured resin cement self-etch	12	17.76	4.67	13.05	27.31	

For pairwise comparisons in shear bond strength, the Bonferroni test was performed (Table 2). The shear bond strength in the light-cured resin cement group was higher than dual-cured resin cement and the pre-heated resin composite groups with statistically significant differences (p=0.000 and 0.003, respectively). No statistically significant difference was found between dual-cured resin cement and the pre-heated resin composite groups (p=1.000; Table 2).

**Table 2:** Pairwise comparison according to Bonferroni test. * Statistically significant at 0.05.

Groups (I)	Groups (J)	Mean diff. (I-J) (Mpa)	SE for the diff.	p-value
Light-cured resin cement	pre-heated resin composite	11.03	2.13	0.000^*^
	Dual-cured self-etch resin cement	8.85	2.13	0.003^*^
pre-heated resin composite	Dual-cured self-etch resin cement	-2.18	2.13	1.000

## Discussion

The development of bonding systems and resins used in adhering ceramics and the increasing interest of patients in aesthetic aspects have led to a major renaissance in cosmetic dental materials [12]. Given the importance of bonding materials and their significant impact on the aesthetics and success of porcelain veneers [13], many resins used in bonding have been developed and improved to achieve a strong bond between porcelain veneers and dental tissues [14], which has led to a significant development of bonding systems to achieve the required durability [15]. The research aimed to evaluate different resins used in luting (IPS e.max) when it came to shear bond strength.

This research demonstrated that different resin cements obtained varying degrees of adhesion to (IPS e.max). Shear bond strength was found to be greatest for the light-cured resin cement while it was significantly lower for the dual-cured resin cement self-etch and Nanofil pre-heated composite. This is attributed to the absence of acid etching for the dual-cured self-etch resin cement, as the preparation in the study sample was limited to the enamel only, and thus the bond strength is lower in the absence of etching [16], so the presence of acid etching increases the bonding surface area, raises its energy and allows the monomers to penetrate the surface of the etched teeth [17].

Studies have shown that preheating the resin at high temperatures improves its physical properties which may lead to increased subsequent polymerization [18] and increases its ease of handling as the higher resin temperatures lead to increased adaptability [19].

Some studies have shown that the temperature of the heated resin decreases by time to about 50% within just the first few minutes of the start of the working time [20], as the higher temperature of the heated composite resin gives the highest bonding strength ​​with the enamel [21] so that when the temperature goes down we lose the ability to improve the bonding with the teeth structure.

The results corroborated with the findings of Stewart et al., who reported that light-cure resin cement showed higher SBS than dual-cure resin cement self-etch [22]. It is recommended to bond ceramic veneers with light-cured resin cement due to its ease of handling, sufficient working time and low solubility [23] and the high success rate of bonding (IPS e.max) veneers with light-cured resin cement [24].

On the other hand, the results were dissimilar to those of Shahin and Katamish, who found that the pre-heated composite resin gave the highest value for SBS [25]. This may be attributed to the differences in materials, the manufacturing company, and working conditions.

In contrast, the results of this study differ from those of Mutlu et al., which evaluated that the pre-heated composite resin gave higher SBS when compared to dual-cured resin cement with a self-etch system [26]. The difference in results may be because, in the present study the (IPS e.max) discs were bonded to enamel, whereas, in that study, the discs were bonded to the dentine. Furthermore, the findings of this study are consistent with those of Lee and Im, who reported a higher bond strength of the light-cured resin cement systems than dual-cured resin cement systems [27].

The study has some limitations, which include a small sample size. As the study was carried out in vitro, that may not simulate the results when studied in clinical because of the different clinical conditions of intra-oral. This research did not include studying color stability, which would give more information about the effect of using different resins when bonding (IPS e.max) on color stability.

## Conclusions

Within the limitations and parameters of the present study, it can be thus concluded that light-cured resin cement is more favorable and ensures a stronger bond to teeth prepared to receive (IPS e.max) veneers and in vitro testing. Although pre-heating composite resins may increase its mechanical proprieties and make it suitable for luting ceramics it may not increase bond strength. Further studies must be done to evaluate the long-term reliability and effectiveness in practice.
